# Exploring the relationship between sleep patterns and depression among Chinese middle school students: a focus on sleep quality vs. sleep duration

**DOI:** 10.3389/fpubh.2024.1383884

**Published:** 2024-06-05

**Authors:** Xinkai Zhang, Zhaobo Dou, Fengying Yang, Lin Luo, Jie Yang

**Affiliations:** ^1^School of Physical Education, Shandong Sport University, Rizhao, China; ^2^Department of Physical Education, China University of Petroleum (East China), Qingdao, China; ^3^School of Sport and Health, Shandong Sport University, Rizhao, China; ^4^School of Physical Education, Guizhou Normal University, Guiyang, China

**Keywords:** adolescent depression, sleep quality, sleep duration, sleep patterns, physical activity

## Abstract

**Objective:**

This study aims to explore the relationship between sleep patterns and depressive symptoms among adolescents, examining variations in depressive symptoms across different sleep qualities, durations, and habits.

**Method:**

A cross-sectional survey was conducted, gathering data from 8,775 Chinese adolescents on their demographics, lifestyle habits, sleep quality and duration, and depressive symptoms. The association between sleep parameters and depressive symptoms was analyzed using multivariate logistic regression.

**Findings:**

The findings reveal a significant correlation between sleep quality/duration and depressive symptoms. Specifically, adolescents with poor sleep quality had higher depressive scores (mean score = 14.62, standard deviation = 5.71), significantly exceeding those with better sleep quality (mean score = 11.54, standard deviation = 4.69). Adolescents with shorter sleep duration also showed significantly higher depressive scores than those with moderate sleep duration. Importantly, adolescents experiencing both poor sleep quality and shorter sleep duration were at a significantly increased risk of depressive symptoms (OR = 4.04, 95% CI: 3.53–4.62, *P* < 0.001). Further analysis indicated that older age and lower family economic status were independent predictors of a higher risk of adolescent depression (OR = 1.22, 95% CI: 1.08–1.38, *P* = 0.001), whereas factors such as gender, ethnicity, residence, being an only child, and parental education levels were not statistically significant.

**Conclusion:**

Among Chinese adolescents, poor sleep quality and shorter sleep duration are independent predictors of higher depressive symptom scores. Adolescents experiencing both of these conditions simultaneously have a significantly increased risk of depressive symptoms. Furthermore, older age and lower family economic status are also significantly related to an increased risk of depression in adolescents. These findings emphasize the importance of improving sleep quality and optimizing sleep duration for the prevention of adolescent depression. They also suggest the need for a comprehensive approach that addresses the multifaceted factors influencing adolescent mental health, including sleep patterns and socioeconomic disparities.

## 1 Introduction

Adolescent mental health issues pose a significant global challenge. The World Health Organization reports that approximately one in seven adolescents worldwide (aged 10–19) suffers from mental disorders, contributing to 13% of the global disease burden in this age group (1). Common mental health challenges include depression and anxiety. Without treatment, adolescent depression can lead to long-term negative outcomes such as substance misuse, unemployment, ongoing psychological struggles, and elevated risks of both mental and physical health issues in later life (2). Studies have shown that these mental health problems are significantly linked to an increased risk of conditions like sleep disturbances, type 2 diabetes, and cardiovascular diseases (3).

Among various factors, sleep, is a critical determinant influencing adolescent depression, with growing evidence pointing to a relationship between diminished sleep duration and heightened depressive symptoms. Insufficient sleep can disrupt the balance of neurotransmitters, like serotonin and norepinephrine, which are essential for emotional regulation (4). Moreover, lack of sleep can intensify the body's stress response, elevating cortisol levels and increasing psychological stress (5). Sleep restriction also affects cognitive functions, particularly those related to processing emotions and decision-making (6).

Previous research has established a significant link between shorter sleep durations and increased depressive symptoms in adolescents. For example, a study on Chinese adolescents found that each hour reduction in sleep duration raised the risk of depressive symptoms by 10% (7). Some experts argue that extending sleep duration could be an effective measure in preventing and managing adolescent depression (8). Yet, this connection has been challenged by recent studies that, after adjusting for confounding factors such as family background and lifestyle habits, found no significant association between short sleep duration and depression (9). This indicates the importance of considering various potential influences when examining the relationship between sleep duration and adolescent mental health (10). However, most of these studies have primarily focused on the relationship between sleep duration and depressive symptoms, while the effects of sleep quality and habits on adolescent mental health have received less attention.

While there has been considerable research on the relationship between sleep duration and adolescent depression, studies exploring the effects of sleep quality and behavioral patterns on mental health are less common. Particularly within the Chinese cultural and social context, the distinct academic pressures and societal expectations faced by middle school students can have a significant impact on their sleep patterns and mental health. This study aims to address this gap by providing a more comprehensive analysis of the relationship between sleep patterns (including duration, quality, and behavioral patterns) and depression among Chinese middle school students. By examining sleep length, quality, and habits, this research aims to understand how these aspects individually and collectively influence adolescent mental health. The outcomes of this study not only offer fresh insights into the sleep patterns-mental health nexus but also contribute to the development of effective mental health interventions, including educational policy formulation and mental health service provision.

## 2 Methods

### 2.1 Participants

This research analyzed original data from the China Education Panel Survey (CEPS), a comprehensive national survey designed to monitor the developmental and educational progress of Chinese middle school students. The aim is to enhance understanding of Chinese adolescents' growth patterns. The study employed a multistage stratified sampling methodology, categorizing sampling units into three levels: counties (or their administrative equivalents), schools, and classes. Initially, counties were classified based on geographic location and the size of the migrant population, with a focus on Shanghai and other areas with significant migrant demographics. Within each selected county, four schools were chosen at random using a probability-proportional-to-size approach. From each school, two seventh-grade classes were picked for participation. The baseline survey encompassed 28 counties, 112 schools, and 224 classes. In these classes, students were asked to fill out a self-administered questionnaire under the guidance of a trained survey team, which was assembled from provincial universities or social science research institutes. To adjust for the uneven probabilities of selection inherent in the sampling strategy, the CEPS team formulated specific survey weights. The data analyzed in this study were collected between 2014 and 2015 and spanned a variety of measures, including demographic information, body mass index, physical activity duration, screen time, sleep duration, sleep quality, and the depression scores of adolescents and their parents. The CEPS survey initially included 9,920 adolescent participants, but the analysis was limited to 8,775 respondents (4,588 males and 4,187 females) who had complete data sets. The study received approval from the Ethics Committee of Renmin University of China. Informed consent was obtained from all participating students and their parents. No rewards were offered to students for participating in the survey.

### 2.2 Key variables and measurement methods

#### 2.2.1 Outcome variable

##### 2.2.1.1 Depression symptoms

This research measured adolescent depressive symptoms using six items adapted from the Patient Health Questionnaire-9 (PHQ-9), as utilized in the China Education Panel Survey (CEPS). These items were selected for their relevance and adaptability in prior studies. The questionnaire's high internal consistency is evidenced by a Cronbach's alpha coefficient of 0.918, affirming the reliability of the measurements. Depression scores were calculated by summing the scores from relevant questions. Consistent with existing literature, participants with scores above the 75th percentile were identified as having depressive symptoms. Thus, the depression score was analyzed both as a continuous and a binary variable, with the latter indicating depressive status.

#### 2.2.2 Exposure variables

##### 2.2.2.1 Sleep duration

Average daily sleep duration, as self-reported by adolescents, was assessed according to the Canadian 24-H Movement Guidelines. For 13-year-olds, the recommended sleep duration is 9–11 h per night, and for those aged 14–17, 8–10 h per night. Sleep duration was categorized based on these age-specific recommendations to analyze the effects of varying sleep durations on adolescent health.

##### 2.2.2.2 Sleep quality

Sleep quality was evaluated through CEPS questions on insomnia, difficulty initiating sleep, frequent nocturnal awakenings, daytime sleepiness, morning fatigue, snoring, bruxism, vivid dreaming, somniloquy, and somnambulism. Participants were classified into good or poor sleep quality groups based on their responses, making sleep quality a categorical variable for analysis.

##### 2.2.2.3 Sleep patterns

Sleep patterns were comprehensively classified into six categories based on quality and duration. Referring to the Canadian 24-H Movement Guidelines, sleep duration was categorized by age into short (< 8 h), moderate (8–10 h), and long (>10 h). Simultaneously, sleep quality was dichotomized as good or poor based on self-reported sleep quality questionnaire scores. By cross-combining these factors, six sleep habit types were obtained: good quality/short duration, poor quality/short duration, good quality/moderate duration, poor quality/moderate duration, good quality/long duration, and poor quality/long duration. This classification aimed to more comprehensively examine the impact of different sleep patterns on depression among adolescents.

#### 2.2.3 Covariates

To comprehensively account for the various factors that may influence adolescent depression symptoms and sleep conditions, the survey questionnaire in this study incorporated background variables from multiple dimensions. These variables included demographic characteristics (e.g., age, gender, ethnicity), family environment (e.g., only-child status, parental education level, family economic status), and lifestyle factors (e.g., physical exercise time, screen time). Furthermore, we assessed adolescents' weight status using body mass index (BMI). As these factors have been shown to be associated with both adolescent sleep and depression symptoms, it is crucial to control for them in the analysis to minimize potential confounding effects.

### 2.3 Statistical analysis

The analysis was performed using Stata 17.0 software (StataCorp LP, College Station, Texas). Categorical variables were summarized with frequencies (%), and continuous variables with means (standard deviations). *T*-tests were applied to assess differences in depression scores across varying sleep qualities. One-way analysis of variance (ANOVA) was employed to evaluate differences in sleep duration and habits. Chi-square tests examined the association between depressive status and variables such as sleep quality, duration, and habits. Logistic regression analysis was utilized to investigate the influence of sleep quality, duration, and habits on the risk of depression among adolescents. The significance level for all statistical tests was set at *P* < 0.05, ensuring that findings were rigorously evaluated for their impact on adolescent depression risk.

## 3 Results

### 3.1 Participant characteristics

This study analyzed the basic characteristics, lifestyle habits, sleep quality and duration, and depressive status of 8,775 Chinese adolescents. The findings (Table 1) indicated an average age of 14.53 years among participants, with a slight male majority (52.28%) compared to females (47.72%). A significant portion of the participants identified as Han ethnicity (91.28%), showing a relatively even split between urban and rural residency. The educational background of parents was predominantly on the lower end, with 56.38% of fathers and 62.88% of mothers having attained an education level of junior high school or below. The bulk of the families fell into the middle-income category (73.46%). In health and lifestyle aspects, the average body mass index (BMI) stood at 18.76. Participants reported spending an average of 156.27 min per week on physical activities and 133.11 min daily on screen time. As for sleep characteristics, 45.20% of adolescents reported experiencing poor sleep quality, while 38.64% had a short sleep duration. The mean depression score was 12.94, with 28.03% of adolescents identified as having depression, highlighting significant mental health concerns within this demographic.

**Table 1:** Characteristics of Chinese adolescents (*n* = 8,775).

	**Mean (SD)/*n* (%)**
Age (year)	14.53 (0.01)
**Gender**	
Girl	4,187 (47.72)
Boy	4,588 (52.28)
**Ethnicity**	
Han	8,010 (91.28)
Non-Han	765 (8.72)
**Residence**	
City	4,195 (47.82)
Rural	4,579 (5,218)
**Single child**	
Yes	3,938 (44.88)
No	4,837 (55.12)
**Father's highest education**	
≤ Junior middle school	4,947 (56.38)
Senior middle school or vocational school	2,377 (27.09)
≥College	1,451 (16.54)
**Mother's highest education**	
≤ Junior middle school	5,518 (62.88)
Senior middle school or vocational school	2,034 (23.18)
≥College	1,223 (13.94)
**Perceived household economic status**	
Lower income	1,806 (20.62)
Middle class	6,434 (73.46)
Wealthy	518 (5.91)
BMI (kg/m^2^)	18.76 (0.05)
PA	156.27 (2.21)
ST	133.11 (1.81)
**Sleep quality**	
Poor	3,993(45.20)
Good	4,782(54.50)
**Sleep duration**	
Short	3,391 (38.64)
Normal	5,303 (60.43)
Long	81 (0.92)
**Sleep patterns**	
Poor quality short duration	1,770 (2-0.17)
Good quality short duration	1,621 (18.47)
Poor quality moderate duration	2,192 (24.98)
Good quality moderate duration	3,111 (35.45)
Poor quality long duration	31 (0.35)
Good quality long duration	50 (0.57)
Depression score	12.94 (0.06)
**Depression status**	
No	6,315 (71.97)
Yes	2,460 (28.03)

### 3.2 Comparison of depression scores and status among adolescents with different sleep qualities, sleep durations, and sleep patterns

The analysis (Table 2) reveals significant associations between sleep quality, sleep duration, and depressive scores and status. Adolescents with superior sleep quality exhibited lower depression scores (mean score = 11.54, standard deviation = 4.69) compared to their counterparts with inferior sleep quality, who had higher depression scores (mean score = 14.62, standard deviation = 5.71). In terms of sleep duration, those adhering to a normal sleep schedule reported lower depression scores than those experiencing either shorter or longer sleep durations. Additionally, an analysis combining sleep quality and duration showed that adolescents experiencing both poor sleep quality and short sleep duration recorded the highest depression scores (mean score = 15.58, standard deviation = 5.94), underscoring the compounded impact of suboptimal sleep patterns on adolescent mental health.

**Table 2:** Comparing adolescent depressive scores and status across various sleep quality, duration, and habits.

	**Depression score**		**Depression status**		
	**Mean (SD)**	***t*** **/** ***F***	**No**	**Yes**	χ^2^
**Sleep quality**					
Good	14.62 (5.71)	< 0.001	2,423 (38.37)	1,157 (63.82)	< 0.001
Poor	11.54 (4.69)		3,892 (61.63)	890 (36.18)	
**Sleep duration**					
Short	13.89 (5.71)	< 0.001	2,244 (35.53)	1,147 (46.63)	< 0.001
Normal	12.36 (5.10)		4,009 (63.48)	1,294 (52.60)	
Long	11.69 (5.28)		62 (0.98)	19 (0.77)	
**Sleep patterns**					
Poor quality short duration	15.58 (5.94)	< 0.001	971 (15.38)	799 (32.48)	< 0.001
Good quality short duration	12.05 (4.83)		1,273 (20.16)	348 (14.15)	
Poor quality moderate duration	13.85 (5.39)		1,434 (22.71)	758 (3,081)	
Good quality moderate duration	11.30 (4.60)		2,575 (40.78)	536 (21.79)	
Poor quality long duration	13.90 (5.53)		18 (0.29)	13 (0.53)	
Good quality long duration	10.32 (4.66)		44 (0.70)	6 (0.24)	

### 3.3 Relationship between sleep quality, sleep duration, and adolescent depression status

To further investigate the relationship between sleep parameters and adolescent depression status, we constructed a multivariable logistic regression model, taking into account the influence of other relevant variables (Table 3). Unlike the preliminary analysis in Table 2, this model focuses on the independent associations between variables, revealing the impact of sleep quality and duration on depression risk after controlling for factors such as age, gender, ethnicity, place of residence, only-child status, parental education levels, and family economic status.

**Table 3:** The relationship between sleep quality, sleep duration, and depressive status.

	**OR**	**95% CI**	***P*-value**
**Sleep quality (CG: Poor)**			
Good	0.37	0.4–0.41	< 0.001
**Sleep duration (CG: Normal)**			
Short	1.51	1.37–0.67	< 0.001
Long	0.85	0.49–1.46	0.565
Age (year)	1.09	1.01–1.17	0.018
**Gender (CG: Girl)**			
Boy	0.97	0.88–1.07	0.616
**Ethnicity (CG: Han)**			
Non-Han	0.92	0.78–1.09	0.380
**Residence (CG: City)**			
Rural	0.92	0.82–1.03	0.197
**Single child (CG: Yes)**			
No	1.06	0.95–1.19	0.257
**Father's highest education (CG:** ≤ **Junior middle school)**			
Senior middle school or vocational school	0.95	0.83–1.09	0.529
≥College	0.99	0.81–1.21	0.938
**Mother's highest education (CG:** ≤ **Junior middle school)**			
Senior middle school or vocational school	0.95	0.82–1.09	0.507
≥College	0.96	0.78–1.18	0.729
**Perceived household economic status (CG: Middle class)**			
Lower income	1.22	1.08–1.38	0.001
Wealthy	0.90	072–1.12	0.351
BMI (kg/m^2^)	1.00	0.99–1.01	0.908
PA	0.99	0.99–1.00	0.117
ST	1.00	1.00–1.00	< 0.001

CG, control group.

The results demonstrate that good sleep quality significantly reduces the risk of depression (OR = 0.37, 95% CI: 0.30–0.44, *P* < 0.001), while insufficient sleep duration is associated with an increased risk of depression (OR = 1.51, 95% CI: 1.37–1.67, *P* < 0.001). Long sleep duration was not found to have a significant effect on depression status (OR = 0.85, 95% CI: 0.49–1.46, *P* = 0.565). Additionally, the regression analysis revealed that lower family economic status is significantly correlated with an increased risk of depression among adolescents (OR = 1.22, 95% CI: 1.08–1.38, *P* = 0.001), highlighting the crucial role of socioeconomic factors in mental health.

Compared to the descriptive analysis in Table 2, logistic regression provides quantitative estimates (OR values) of the impact of each variable on depression and their precision (confidence intervals), enabling a more comprehensive understanding of the relationship between sleep and depression.

### 3.4 Relationship between sleep patterns and adolescent depression status

Multivariable logistic regression analysis was conducted to examine the impact of sleep patterns on adolescent depression status. According to the findings (Table 4), adolescents exhibiting both poor sleep quality and short sleep duration face a significantly higher risk of depression (OR = 4.04, 95% CI: 3.53–4.62, *P* < 0.001). Conversely, good sleep quality correlates with a reduced risk of depression, even when sleep duration is short (OR = 1.37, 95% CI: 1.17–1.59, *P* < 0.001). Further analysis shows that while variables such as parental education level and place of residence do not significantly affect depression status in adolescents, lower family economic status remains a significant predictor of increased depression risk (OR = 1.22, 95% CI: 1.08–1.38, *P* = 0.001), underscoring the significant role socioeconomic factors play in the mental health of adolescents.

**Table 4:** The relationship between sleep patterns and depressive status.

	**OR**	**95% CI**	***P*-value**
**Sleep patterns (CG: Good quality moderate duration)**			
Poor quality short duration	4.04	3.53–4.62	< 0.001
Good quality short duration	1.37	1.17–1.59	< 0.001
Poor quality moderate duration	2.47	2.17–2.81	< 0.001
Poor quality long duration	2.88	1.39–5.95	0.004
Good quality long duration	0.58	0.24–1.38	0.223
Age (year)	1.09	1.01–1.17	0.019
**Gender (CG: Girl)**			
Boy	0.97	0.88–1.07	0.602
**Ethnicity (CG: Han)**			
Non-Han	0.92	0.78–1.09	0.386
**Residence (CG: City)**			
Rural	0.92	0.82–1.03	0.194
**Single child (CG: Yes)**			
No	1.06	0.95–1.19	0.253
**Father's highest education (CG:** ≤ **Junior middle school)**			
Senior middle school or vocational school	0.95	0.83–1.09	0.529
≥College	0.99	0.81–1.21	0.948
**Mother's highest education (CG:** ≤ **Junior middle school)**			
Senior middle school or vocational school	0.95	0.82–1.09	0.497
≥College	0.96	0.78–1.18	0.737
**Perceived household economic status (CG: Middle class)**			
Lower income	1.22	1.08–1.38	0.001
Wealthy	0.90	072–1.12	0.349
BMI (kg/m^2^)	1.00	0.99–1.01	0.937
PA	0.99	0.99–1.00	0.113
ST	1.00	1.00–1.00	< 0.001

CG, control group.

## 4 Discussion

This study aimed to examine the relationship between sleep patterns and the status of depression among adolescents, recognizing adolescence as a pivotal phase for mental health development and sleep as a critical physiological necessity with significant implications for adolescent mental health. The findings underscore the significance of both sleep quality and sufficient sleep duration in preventing adolescent depression.

The results highlight that poor sleep quality is linked to an increase in depression among adolescents, aligning with Gregory and O'Connor's (11) observation of a robust link between sleep disturbances and depression. Consistent with prior research (12, 13), poor sleep quality can disrupt emotional regulation, cognitive function, and amplify the stress response, all identified as depression risk factors (14, 15). Moreover, this study extends the understanding by emphasizing the critical role of sleep quality and the compound negative effects of poor sleep quality and short sleep duration on adolescent mental health.

Furthermore, this study confirmed the strong correlation between short sleep duration and an increased risk of depression. This finding aligned with Shochat et al. (16), who highlighted the negative impact of insufficient sleep on emotional well-being and depression among adolescents. Short sleep duration is tied to fatigue, diminished concentration, and mood swings, symptoms commonly associated with depression (17, 18).

The lack of a significant correlation between long sleep duration and depression status suggests a more complex relationship between excessive sleep and depression, highlighting the critical role of both sleep quality and adequate sleep duration in preventing adolescent depression. While prior research has primarily focused on sleep duration, our findings suggest that sleep quality is an equally important factor in preventing adolescent depression. Even when sleep duration was short, good sleep quality was associated with a lower risk of depression. This highlights the need for a more comprehensive approach to promoting healthy sleep patterns among adolescents, considering both sleep quality and duration.

Furthermore, this study reveals significant differences in depression across various sleep quality, duration, and habit categories. Adolescents with poor sleep quality and short sleep duration exhibited the highest depressive scores, while those with good sleep quality and moderate sleep duration had the lowest scores. These findings underscore the importance of considering both sleep quality and duration when assessing adolescent mental health and developing intervention strategies. Traditional approaches that solely focus on sleep duration may overlook the crucial role of sleep quality in preventing depression.

Additionally, the study explored the interaction between sleep patterns and factors such as age, gender, ethnicity, residence, only-child status, parental education, and family economic status on depression. The literature indicates that these sociodemographic elements have nuanced impacts on adolescent mental health. Gender differences, for instance, are significant, with females more likely to experience depression (19). Economic stress and parental education levels are also closely tied to mental health outcomes, affecting the prevalence of depression among adolescents (20, 21). However, in this study, factors such as gender, ethnicity, residence, being an only child, and parental education levels were not independent predictors of depression risk, contrary to some previous findings (22). These discrepancies may be attributed to differences in study populations, measurement tools, and socio-cultural contexts, warranting further investigation. These interactions suggest the need for a comprehensive approach to understand and address adolescent depression fully, considering both sleep patterns and socioeconomic factors.

The study's limitations include its reliance on a non-diverse sample, as it only included seventh-grade students, which may not fully represent the entire middle school population, particularly ninth-grade students facing academic pressure related to graduation. Future research should expand the sample range to include students from grades seven to nine, thereby improving the generalizability of the results. Additionally, the use of self-reported data may introduce bias. To address this, future studies should incorporate objective measures of sleep and depression.

It is worth noting that there is currently no unified standard for classifying adolescent sleep patterns. Although the classification method employed in this study has a basis in the literature, it still requires further validation. Future research could explore more scientific and refined ways of categorizing sleep patterns to more accurately characterize adolescent sleep patterns and their relationship with mental health.

Despite these limitations, as an exploratory study, this research has taken an important step toward revealing the relationship between sleep patterns and depression among Chinese adolescents, laying the foundation for subsequent validational studies with larger, more diverse samples. Moreover, examining factors such as social media use, academic pressure, and employing longitudinal designs could further enrich our understanding of the sleep-depression nexus in adolescents.

The findings of this study advocate for enhancing awareness of the importance of sleep in adolescent mental health among educators and parents. Implementing strategies to foster regular sleep patterns and minimize nighttime screen exposure can support adolescent mental health and overall wellbeing, underscoring the vital role of sleep patterns in preventing depression. By addressing the identified limitations and expanding the scope of future research, we can gain a more comprehensive understanding of the complex interplay between sleep and mental health in adolescents, ultimately informing the development of effective interventions and policies to promote the wellbeing of this vulnerable population.

## 5 Conclusion

This research explored the sleep patterns of 8,775 Chinese adolescents and their association with depression status. The findings clearly demonstrate that poor sleep quality and shorter sleep duration are significantly correlated with higher depressive scores among adolescents. Importantly, adolescents experiencing both poor sleep quality and shorter sleep duration were at a markedly increased risk of depression (OR = 4.04, 95% CI: 3.53–4.62, *P* < 0.001). The study also emphasizes the influence of sociodemographic elements on adolescent depression, with older age and lower family economic status being independent predictors of a higher risk (OR = 1.22, 95% CI: 1.08–1.38, *P* = 0.001). However, factors such as gender, ethnicity, residence, being an only child, and parental education levels were not found to be statistically significant in this study.

These insights highlight the critical need to enhance sleep quality and optimize sleep duration as strategies for preventing depression among adolescents. They also serve as a reminder for parents, educators, and healthcare professionals to consider the multifaceted factors affecting adolescent mental health, advocating for a holistic approach to support the wellbeing of young individuals. Interventions targeting sleep patterns, as well as those addressing socioeconomic disparities, may prove beneficial in reducing the risk of depression among Chinese adolescents.

## Data availability statement

The datasets presented in this study can be found in online repositories. The names of the repository/repositories and accession number(s) can be found at: http://ceps.ruc.edu.cn/.

## Ethics statement

Ethical review and approval was not required for the study on human participants in accordance with the local legislation and institutional requirements. Written informed consent from the participants or participants' legal guardian/next of kin was obtained in accordance with the national legislation and the institutional requirements.

## Author contributions

XZ: Conceptualization, Data curation, Formal analysis, Methodology, Validation, Writing – original draft. ZD: Data curation, Resources, Writing – original draft. FY: Data curation, Resources, Writing – original draft. LL: Methodology, Validation, Writing – review & editing. YJ: Project administration, Supervision, Validation, Writing – review & editing.
